# Coronary bioresorbable stents: Non-invasive quantitative evaluation of intra- and juxta-stent plaque composition—A computed tomography longitudinal study

**DOI:** 10.1371/journal.pone.0268456

**Published:** 2022-10-13

**Authors:** Evguenia Zdanovich, Samer Mansour, Louis-Mathieu Stevens, Charbel Naim, Daniel Juneau, Alexandre Semionov, Carl Chartrand-Lefebvre

**Affiliations:** 1 Radiology Department, Centre Hospitalier de l’Université de Montréal (CHUM), Montreal, Quebec, Canada; 2 Centre de Recherche du Centre Hospitalier de l’Université de Montréal (CRCHUM), Montreal, Quebec, Canada; 3 Cardiology Department, CHUM, Montreal, Quebec, Canada; 4 Cardiac Division of the Surgery Department, CHUM, Montreal, Quebec, Canada; 5 Nuclear Medicine Department, CHUM, Montreal, Quebec, Canada; 6 Radiology Department, McGill University Health Center (MUHC), Montreal, Quebec, Canada; Medical University Innsbruck, AUSTRIA

## Abstract

**Purpose:**

Coronary bioresorbable stents (BRS) do not produce blooming artifacts on computed tomography (CT), in contrast to metallic stents, as they are made of a bioresorbable polymer and are radiolucent. They allow to evaluate the coronary plaque beneath. The low-attenuation plaque (LAP) suggests plaque vulnerability and is CT assessable. The aim of our study was to show the possibility of a non-invasive CT evaluation of the volume and the LAP composition of the intra- and juxta-stent plaque.

**Methodology:**

In our prospective longitudinal study, we recruited 27 consecutive patients (35 BRS stents total; mean age 60 +/- 9 years) with bioresorbable stents for a 256-slice ECG-synchronized CT evaluation at 1- and 12-months post stent implantation. Total plaque volume (mm3), absolute and relative (%) LAP volume per block in the pre- intra- and post-stent zones were analyzed; comparison 1- and 12-months post-implantation of BRS. Changes in the previously mentioned variables were assessed by the mixed effects models with and without spline, which also accounted for the correlation between repeated measurements.

**Results:**

Our block or spline model analysis has shown no significant difference in plaque or absolute LAP volumes in pre- intra- and post-stent zones between 1 and 12 months. Interestingly, % LAP volume increases near-significantly in the distal block of the intrastent at 12-mo follow-up (from 23.38 ± 1.80% to 26.90 ± 2.22% (increase of 15%), p = 0.052).

**Conclusion:**

Our study demonstrates the feasibility of the repeated non-invasive quantitative analysis of the intrastent coronary plaque and of the in-stent lumen by CT scan.

## Introduction

Scaffold of coronary bioresorbable stents (BRS) is made of a bioresorbable polymer rather than metal. Once implanted in the artery, BRS progressively degrade and disappear within 2–3 years [1, 2]. BRS were developed to prevent permanent jailing of stented coronary segments, as it is the case with metallic stents, with the theoretical advantage of eventual normalization of adaptive vascular remodeling mechanisms [3]. Moreover, BRS do not produce significant artifacts on computed tomography (CT), in contrast to metallic stents [4–6]. BRS can be identified on CT by the platinum markers located at each extremity, while the rest of the stent is radiolucent [5]. Therefore, this novel scaffold platform allows noninvasive CT assessment of intrastent lumen, free of any metallic artefact.

In native coronary arteries, beyond the diagnosis of luminal stenosis, CT is useful for detection and volume quantification of atherosclerotic plaque. Furthermore, CT allows plaque characterization through identification of high-risk plaque features. Plaques can be characterized according to their CT attenuation by extraction of low-attenuation plaque content, i.e. lipid-content fraction, defined as attenuation values ≤ 30 Hounsfield units (HU). Low-attenuation plaque content has been associated with higher incidence of acute coronary events [7, 8].

In patients with metallic stents, intrastent imaging is performed using intracoronary invasive procedures, such as intravascular ultrasound (IVUS) and optical coherence tomography (OCT) [9–12]. These techniques allow assessment of atherosclerotic lesion characteristics before stent implantation, as well as intrastent lumen and plaque evolution after implantation. However, blooming artifacts from metal struts [4] are precluding noninvasive CT imaging of intrastent plaques. We have hypothesized that CT can be effectively used for noninvasive assessment of the burden, high-risk features and evolution of intrastent atherosclerotic plaques in patients with BRS.

The aim of our study is to demonstrate the feasibility of a noninvasive CT evaluation ☯dataset, author name: Evguenia Zdanovich, dataset title: “Liste patient REABSORB-CT—8 août 2017_EZ”, data repository: Macintosh HD/Users/Eugenie/Documents/Maîtrise EZ/MAIN PROJECT/Liste des patients, version 2.0, year: 2017, global persistent identifier: created- August 8, 2017 at 3:36 PM] of volume and low-attenuation content of the intrastent coronary plaques, as well as plaques at the edges of the bioresorbable stents.

## Materials and methods

### Study design

This is a prospective cohort study that recruited consecutive patients with BRS for a 256-slice ECG-synchronized CT assessment at 1 and 12 months after stent implantation.

### Ethical approval

The Institutional Review Board of the CHUM (Centre hospitalier de l’Université de Montréal) approved the protocol (approval 15.397). All subjects signed an informed consent form.

### Study patients

The present study is part of in the ReABSORB registry [13]. ReABSORB is a prospective, nonrandomized, observational registry of 125 patients treated with an everolimus (Afinitor)-eluting BRS (ABSORB^TM^, version 1.1) at Centre Hospitalier de l’Université de Montréal in Montreal (CHUM) and Cité-de-la-Santé Hospital in Laval [13]. In the ReABSORB study, clinical follow-up was conducted at 1, 6, and 12 months, then yearly up to 4 years. The present study is a pilot study, nested in the ReABSORB registry. Sample of the pilot study was decided at 25 ReABSORB consecutive participants. The inclusion criteria of the present study were being a participant in the ReABSORB study, with 18 years of age and older. Exclusion criteria comprised renal impairment, adverse reaction to intravenous contrast agents and pregnancy. Twenty-seven consecutive patients were prospectively enrolled in the present study (36 BRS) (mean age 59.7 +/- 8.6 yo; 17 males, 59.3 +/- 8.9 yo; 10 females, 60.5 +/- 8.3 yo).

**Fig 1:**
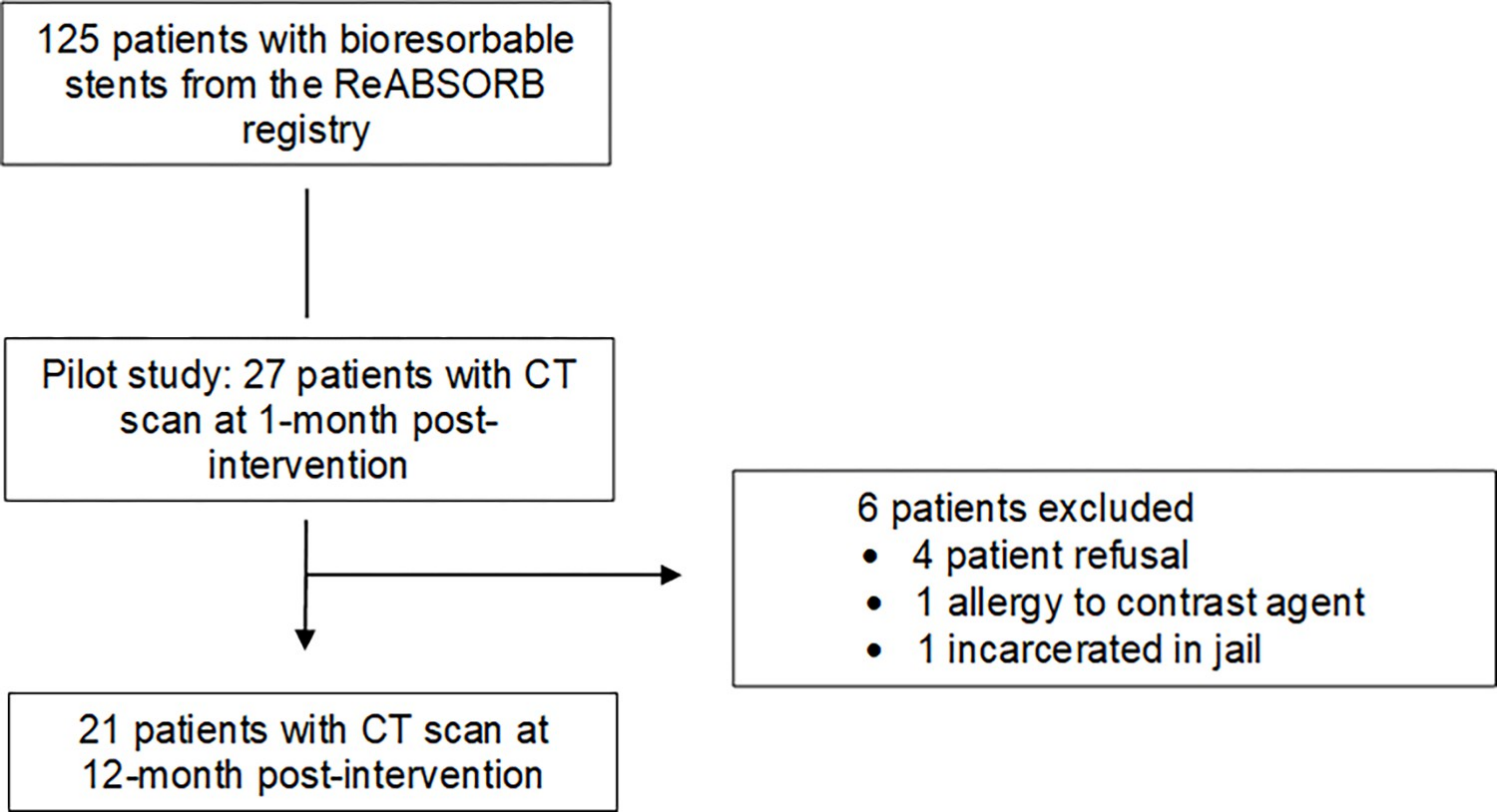
Flow chart of patients bioresorbable coronary stents through the study. All participants were recruited consecutively from the ReABSORB registry. Six participants were excluded. A total of 27 participants underwent a CT evaluation, then 21 underwent a 12-month follow CT.

Two CT scans were planned for each patient, at 1 and 12 months after stent implantation, in order to occur as part of the predetermined clinical follow-up schedule. One-month scan was chosen as to be the closest to implantation. The 12-month follow-up scan was chosen in order to be comparable to time intervals from stent implantation reported in other studies [14], as well as to reduce loss to follow-up. Twenty-seven patients (35 stents) had a CT scan at 1-month post-intervention. Among those 27 patients, 21 (26 stents) were scanned a second time approximatively at 12 months post-implantation. Six patients of the initial 27 patients were not scanned at 12 months (4 patient refusal, 1 allergy to contrast agent, 1 incarcerated in jail) (Fig 1).

#### Stent implantation protocol

BRS implantation was performed at the cardiologist’s discretion [13] and the following procedure was usually followed: (1) to select appropriate BRS size and adequate vessel preparation, vessel sizing was performed with QCA, OCT or IVUS; (2) a balloon at least 5 mm shorter than the chosen BRS length was deployed in predilation preparation, where balloon diameter was identical to the reference vessel diameter; (3) slow deployment of BRS, respecting reference vessel diameter, was conducted in 2-atm increments every 5 seconds until the BRS was completely deployed at a maximal pressure of 12–18 atm; and (4) to prevent BRS non-apposition, postdilation was performed with a noncompliant balloon with ≥0.25–0.5mm higher diameter than the BRS [13].

The struts of ABSORB BRS are made of bioresorbable poly-L-lactide and are 150-mm in width. Those struts are covered with a 7-mm layer of poly-D,L-lactide bioresorbable material and elute everolimus (Novartis, Basel, Switzerland). These stents were available since 2012 through a special access program and were approved for clinical practice in Canada since July 14, 2016 [13].

### CT imaging

#### Patient preparation

Prior to scan, patients received 0.4 mg nitroglycerin sublingually and 25–75 mg metoprolol per os, if their heart rate was > 60 beats per minute (bpm), unless contraindicated.

#### CT acquisition protocol

All examinations were performed on a 256-slice CT scanner (Brilliance iCT, Philips Healthcare, Best, Netherlands). Prospective ECG-gating was used for heat rates ≤ 70 bmp and retrospective ECG-gating for higher heart rates.

#### Contrast administration protocol

Patients received one of the following contrast agents: iopamidol (370 mg/mL, Isovue 370, Bracco Imaging, Montreal, Canada), iodixanol (320 mg/mL, Visipaque 320, GE Healthcare Canada Inc., Mississauga, Ontario, Canada); or iohexol (350 mg/ml, Omnipaque 350, GE Healthcare Canada Inc., Mississauga, Ontario, Canada) (Table 3). Contrast agent was administered with a power injector at a flow rate of 5 ml/sec.

#### CT image reconstruction and postprocessing

Axial reconstruction was done using a medium-smooth kernel (XCB, Philips Healthcare, Cleveland, OH, USA) with a slice thickness of 0.8 mm. Iterative reconstruction (IR) was performed with a hybrid statistical algorithm (Philips iDose, Philips Healthcare, level 3). Post-processing of images was performed by TeraRecon (Aquarius Intuition version 4.4.12, TeraRecon Headquarters, Forster City, CA, USA).

#### Radiation dose

Effective radiation dose estimate was obtained by multiplying total dose-length product (DLP) with a conversion coefficient of 0.014 mSv /cm^-1^*mGy^1^.

### CT imaging analysis

**Fig 2:**
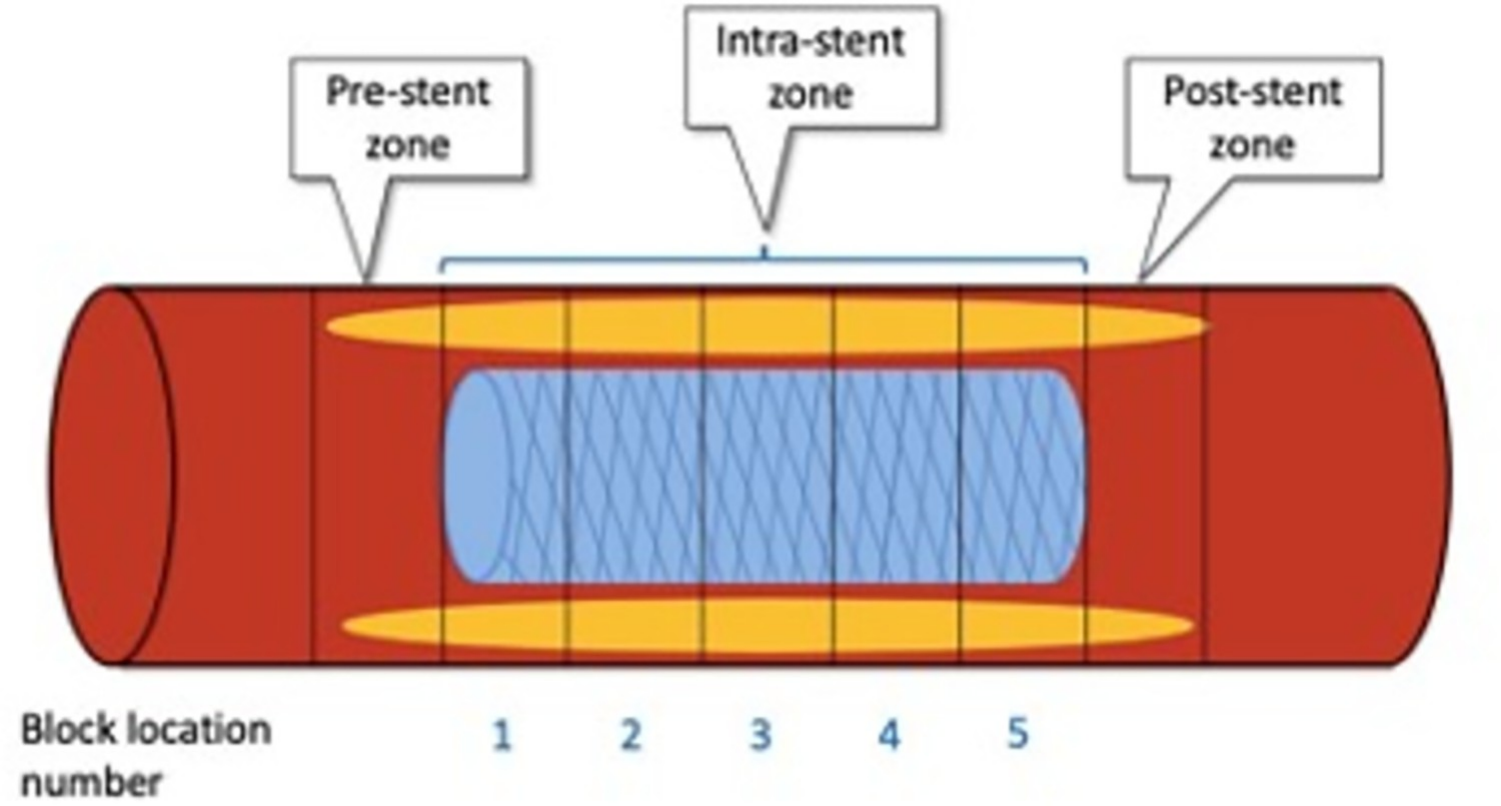
Pre-, intra- and post-stent zones composition with 5-mm blocks (vertical rectangles). Pre-stent zone is the most proximal zone, while post-stent zone is the most distal. Bioresorbable stents also have 2 platinum indicators located at the extremities of the stent in the intrastent blocks numbered 1 and 5. 2016 © ☯Omar Arfa and Evguenia Zdanovich].

For coronary intra-stent and edge plaque analysis, 3 coronary zone locations were defined as follows: pre-, intra- and post-stent zones (Fig 2). Every zone was divided into blocks of 5 mm length following the long axis of the arteries. Pre-stent and post-stent zones were represented by one block each. The intra-stent zone had one to five blocks, depending on the length of the stent. The intra-stent block count started at the platinum indicator of the proximal stent edge and ended close to the indicator of the distal edge.

The coronary segments were described according to the nomenclature established by the American College of Cardiology/American Heart Association guidelines for coronary angiography 1999 [15].

**Fig 3:**
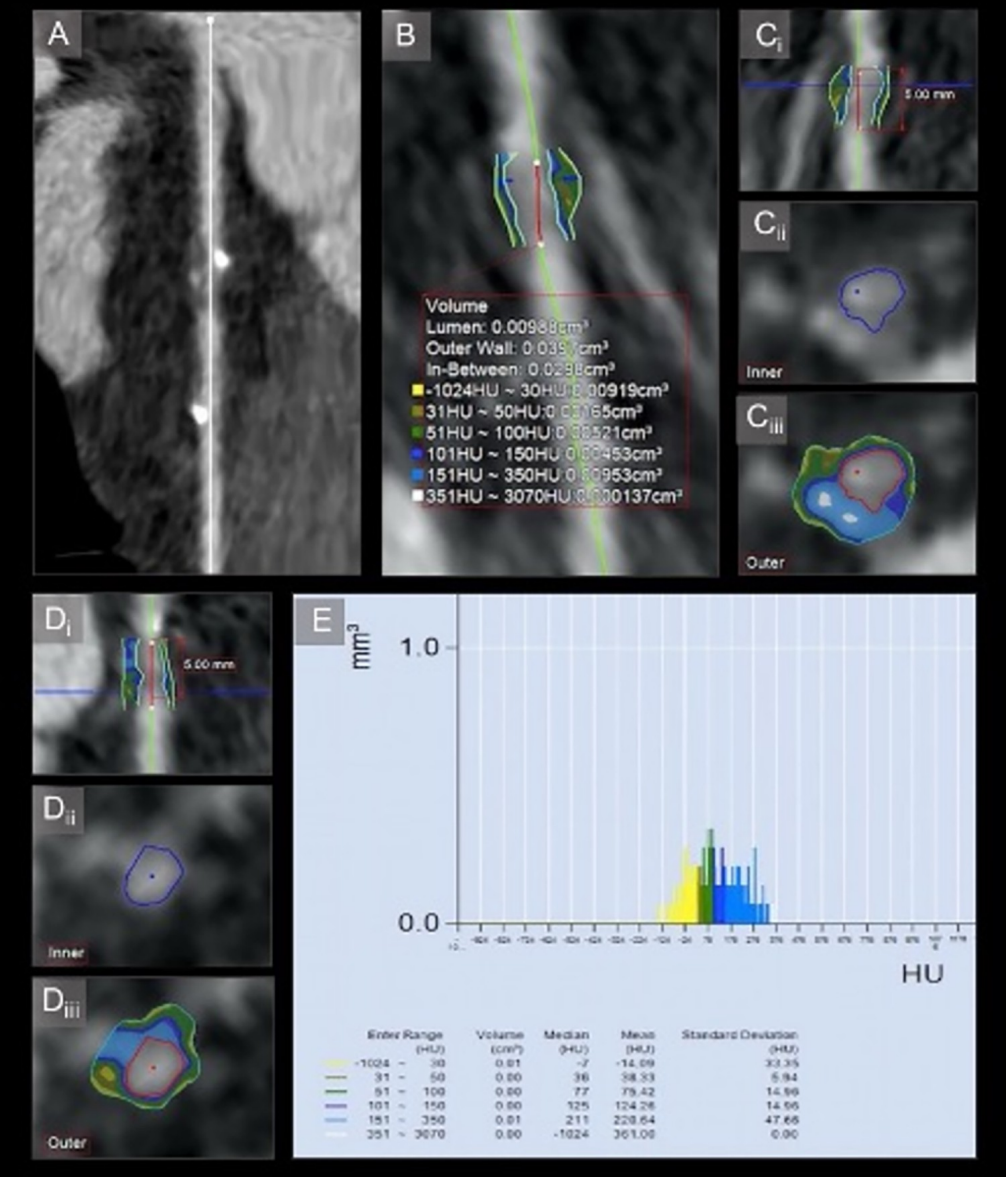
Volumetric plaque analysis. (A) Straightened MPR. There is stenosis at the proximal edge of the BRS in middle left anterior descending coronary artery. (B) Volumetric analysis of plaque HU-stratification. (Ci) Position within the plaque of the axial slice presented in views Cii and Ciii. (Cii) Axial view of lumen in Ci slice. (Ciii) Axial view of the mixed plaque with color-coded LAP and two spotty calcifications. (Di) Position within the plaque of axial slice presented in views Dii and Diii. (Dii) Axial view of lumen in Di slice. (Diii) Axial view of color-coded LAP plaque. (E) Histogram of the plaque composition stratification. LAP (9.19 mm^3^) represents one third of the total plaque volume (29.8 mm^3^). 2016 © ☯Omar Arfa and Evguenia Zdanovich].

Plaque analysis included total plaque volume (mm^3^), absolute LAP volume (mm^3^) and relative LAP volume (%) per block plaque volume in the pre-, intra- and post-stent zones. LAP was defined as plaque component with a <30 Hounsfield units (HU) CT attenuation. Relative LAP volume was defined as absolute LAP volume / total plaque volume (%). LAP volume was detected automatically by software in the 5-mm length coronary blocks following manual tracing of vessel contours (Fig 3). Image postprocessing was performed by a trained operator using a semi-automated software (Aquarius iNtuition 4.4.12, TeraRecon Inc, Foster City, CA, USA). Comparison of results was made between 1- and 12-month post-BRS implantation.

The plaque analyses were performed at the same location in a given coronary artery for both 1- and 12-month scans.

### Statistical analysis

Data was expressed as mean ± standard deviation or median [interquartile range] for continuous variables and frequency (%) for categorical variables. Mixed effects models were used to assess changes in total plaque volume and absolute or relative LAP volumes, and account for the correlation between repeated measurements (MIXED procedures in SAS software, version 9.4; SAS Institute, Cary, NC). Assessed coronary artery vessels and stented regions were divided in 3 zones (pre-stent, intra-stent, and post-stent). The intra-stent zone was further divided in 3 equidistant thirds using piecewise linear regression. P-values <0.05 were considered statistically significant.

## Results

### Study patients

**Fig 4:**
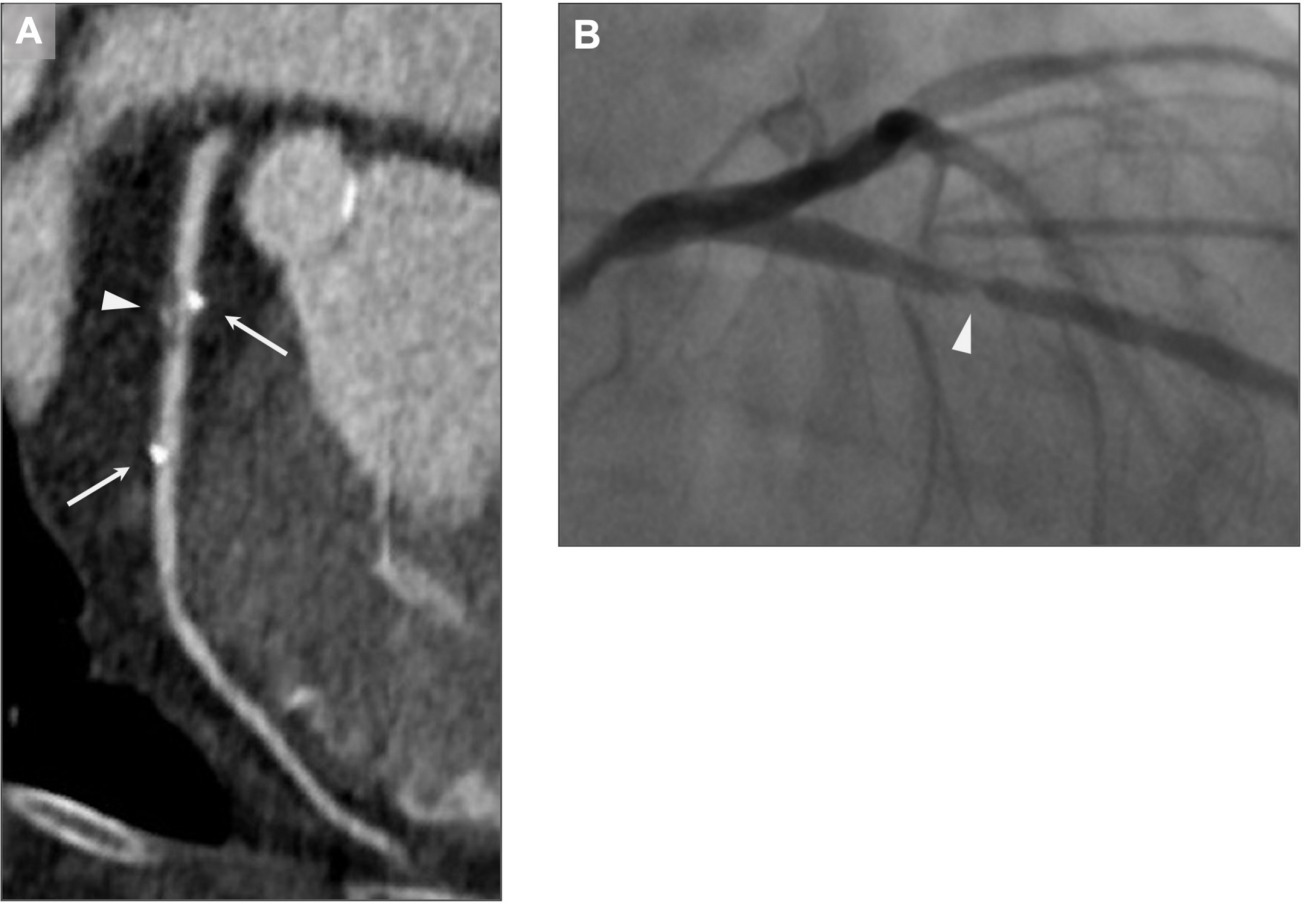
Intra-stent stenosis. A). ECG-gated 256-slice coronary CT angiography 14 months after BRS implantation, in a 61-yo woman. An edge and intra-stent mixed plaque with severe stenosis and positive remodeling is shown (arrowhead). Proximal and distal platinum indicators of the BRS (2.5 x 18 mm) are also visible (arrows). 2016 © ☯Carl Chartrand-Lefebvre and Evguenia Zdanovich]. B) Conventional coronary angiography confirms severe intra-stent stenosis. The patient underwent stenting with an everolimus-eluting metallic stent in the same session. 2016 © ☯Samer Mansour].

The 27 patients had a total of 36 stented coronary artery segments: 25 (69%) in the left coronary artery, and 11 (31%) in the right coronary artery (Table 1). All scanned BRS could be assessed at about 1- and 12-month follow-up. One patient demonstrated an intra-stent stenosis in the LAD, at 12-month scan. This stenosis was subsequently dilated using an everolimus-eluting metallic stent (Fig 4). All other 35 stents were patent at the 1- and 12-month CT scans.

**Table 1:** Stent distribution parameters.

N patients at 1^st^ scan *	27 (100)
N patients at 2^nd^ scan	21 (78)
Total n of stents	36^§^
N of stents at 1^st^ scan	35 (97)
N stents at 2^nd^ scan	26 (72)
Time interval (days) (min-max) **:	
From BRS implantation to 1^st^ scan	35.8 ± 38.4 (7–188)
From BRS implantation to 2^nd^ scan	393.7 ± 45.3 (247–453)
From 1^st^ to 2^nd^ scan	356.7 ± 68.6 (185–446)
N of coronary segments stented by BRS among the whole group of patients	36
Left coronary artery:	
Proximal LAD	7 (19)
Mid LAD	12 (33)
Distal LAD	1 (3)
First diagonal	1 (3)
Proximal Cx	1 (3)
Mid Cx	1 (3)
Distal Cx	1 (3)
1^st^ obtuse marginal	1 (3)
Right coronary artery:	
Proximal RCA	2 (6)
Mid RCA	6 (17)
Distal RCA	2 (6)
PDA	1 (3)
N of stented segments / patient	1.3 ± 0.6 (1–3)
Stent length (mm)	22.4 ± 5.6 (12–28)
Stent diameter (mm)	3.2 ± 0.4 (2.5–3.5)

* Categorical variables are reported as n (%)

** Continuous variables are reported as (mean ± SD) (min-max)

§ One stent imaging was only performed at 12-month as one patient had a second BRS implanted after his 1-month scan of his first BRS. Thus, there are 35 stents at 1-month scan but a total of 36 stents for both scans.

LAD = left anterior descending coronary artery, Cx = circumflex coronary artery, RCA = right coronary artery, PDA = posterior descending coronary artery, SD = standard deviation.

Patient demographics and scanning parameters are described in Tables 2 and 3. Before BRS implantation, lesions were classified based on the ACC/AHA classification of coronary lesions [16]. There were 9 type A lesions (discrete, concentric lesion with smooth contours and absence of thrombus), 11 type B1 lesions and 8 type B2 lesions (lesion type B is tubular, eccentric with some thrombus potentially present, where subtype B1 is determined by one type B characteristic and subtype B2 by ≥2 characterictics), and 1 type C lesion (diffuse and high risk, and is present in tortuous and extremely angulated coronary segments) (Table 4). Most BRS had a length of 18 mm (47%) or 28 (47%) mm, with a mean length of 22.4 ± 5.6 mm (Table 5).

**Table 2:** Patient demographics (N patients = 27).

Male gender *	17 (63)
Age (years) **	59.7 ± 8.5 (41–77)
BMI (kg/m^2^)	27.0 ± 5.7 (18–43)
Risk factors:	
Dyslipidemia	13 (48)
Diabetes	2 (7)
Hypertension	16 (59)
Smoking history	11 (41)
TIA/ stroke	1 (5)

* Categorical variables are reported as n (%)

** Continuous variables are reported as (mean ± SD) (min-max)

n–number of patients, SD—standard deviation, BMI—body mass index, TIA—transient ischemic attack.

**Table 3:** Scan parameters.

		1-mo	12-mo
Prospective ECG gating *		26 (96)	19 (95)
Retrospective ECG-gating		1 (4)	1 (5)
100 kV Voltage		0	6 (29)
120 kV Voltage		27 (100)	15 (71)
Current (mA)(min-max)**		828.9 ± 125.9 (551–1199)	775.4 ± 109.6 (496–1000)
DLP scan (mGy•cm)		413.9 ± 220.9 (226–1456)	372.8 ± 217.4 (164–1214)
Effective dose scan *** (mSv)		5.8 ± 3.1 (3–20)	5.2 ± 3.0 (2–17)
Z-coverage (mm)		152.7 ± 19.5 (125–192)	166.4 ± 21.2 (125–191)
Prescan metoprolol		16 (59)	14 (88)
Prescan nitroglycerin		24 (92)	19 (100)
Contrast type*:			
	Iodixanol (320 mg I/mL)	12 (46)	6 (30)
	Iopamidol (370 mg I/mL)	11 (42)	13 (65)
	Iohexol (350 mg I/mL)	3 (12)	1 (5)

* Categorical variables are reported as n (%)

** Continuous variables are reported as (mean ± SD) (min-max)

*** The effective radiation dose = total DLP•conversion coefficient k, where

k = 0.014 mSv•mGy^−1^•cm^−1^; n—number of patients, SD- standard deviation; mg I/mL—mg of iodine per milliliter.

**Table 4:** Pre-implantation lesion characteristics as assessed on conventional angiography (N stents = 29).

Lesion length (mm)*		13.2 ± 4.6 (8–24)
Calcifications		2 (7)
Lesion type***		
	A	9 (31)
	B1	11 (38)
	B2	8 (28)
	C	1 (3)
Vessel angulation**		
	< 45°	26 (90)
	45°-90°	3 (10)
Bifurcations		5 (17)

* Continuous variables are reported as (mean ± SD) (min-max)

** Categorical variables are reported as n (%)

*** Based on ACC/AHA classification of coronary lesions.

Lesion type A is discrete, concentric with smooth contours and absence of thrombus. Lesion type B is tubular, eccentric with some thrombus potentially present, where subtype B1 is determined by one type B characteristic and subtype B2 by ≥2 characterictics. Lesion type C is diffuse and high risk, and is present in tortuous and extremely angulated coronary segments).

**Table 5:** Description of the different sizes of BRS in our study (36 stents total).

Scaffold design	BRS diameter (mm)	BRS length (mm)		
		12	18	28
Small	2.5	0	4	2
	3.0	0	6	5
Medium	3.5	2	7	10

The numbers in the matrix represent the number of stents

### CT plaque analysis

Plaque analysis was performed on a per-block basis. There were 177 (88%, 177/201) assessable blocks at 1-month post-implantation out of a total of 201 blocks. For matched comparison at 12 months, 132 (66%, 132/201) blocks were assessable out of 201 blocks for a total of 309 block assessments (77%, 309/402) for both scans. Ninety-three (23%, 93/402) blocks were non-assessable for longitudinal imaging analysis. We were not able to assess all the blocks (93 blocks not assessed in total) for following reasons: absence of a second scan (6 patients, 11 stents, 51 blocks); absence of the first scan (1 patient, 1 stent, 7 blocks); stent overlap (4 patients, 10 stents, 21 blocks); image artefacts (8 patients, 8 stents, 9 blocks); blocks shorter than 5 mm (4 patients, 4 stents, 5 blocks).

#### Plaque volume analysis

*Plaque volume using the 5mm-long blocks model of segmentation*. Table 6 shows mean plaque volume of the pre-, intra- and post-stent blocks after 1- and 12-month follow-up, according to the 5mm-long model of plaque segmentation. Intrastent block location is divided from proximal to distal blocks. There is a slight non-significant decrease in plaque volume from 1- to 12-month follow-ups in all locations. More distal plaques show greater decrease in volume in comparison to more proximal plaques, at 1- and 12-month follow-ups.

**Table 6:** Plaque volume at 1- and 12-month follow-ups by block location (pre-, intra 1-, 2-, 3-, 4-, 5-, and post-stent blocks).

Block location^ψ^	Plaque volume (mean ± SD, mm^3^) (# blocks) at 1-mo scan	Plaque volume (mean ± SD, mm^3^) (# blocks) at 12-mo scan	Volume variation (%) between scans	p-value
**Pre-stent**	30.38 ± 11.83 (28)	26.06 ± 10.81 (18)	-14	0.803
**Intra-stent 1**	31.85 ± 12.06 (33)	31.63 ± 12.67 (25)	-1	0.627
**Intra-stent 2**	32.11 ± 12.95 (34)	30.23 ± 11.25 (26)	-6	0.722
**Intra-stent 3**	29.58 ± 10.72 (30)	27.32 ± 11.51 (24)	-8	0.567
**Intra-stent 4**	31.53 ± 12.01 (14)	29.23 ± 15.81 (12)	-7	0.286
**Intra-stent 5**	26.26 ± 5.67 (9)	22.00 ± 6.94 (7)	-16	0.324
**Post-stent**	24.24 ± 11.08 (29)	22.73 ± 10.13 (20)	-6	0.748

^ψ^A total of 27 patients, 36 stents and 309 blocks were analyzed.

Note: The intra-stent blocks are being enumerated (1 to 5) from proximal to distal edge of the stent. Some stents are only 1-,2-,3-,4-blocks long (see stent dimensions in Table 5). –intra-group p-values are represented in the blue bubbles. P-values are obtained by the ANOVA analysis with Bonferroni correction.

*Plaque volume using the tertiles model of segmentation*. Table 7 demonstrates plaque volume in the pre-, intra- and post-stent locations on 1- and 12-month scans. Intra-stent plaques are classified into proximal, median and distal tertiles. There is no significant change in plaque volumes from 1- to 12-month follow-ups in all locations.

**Table 7:** Plaque volume at 1- and 12-month follow-ups by plaque location (pre-, 1^st^, 2^nd^, 3^rd^ intra-stent tertiles, and post-stent plaque).

Plaque location^ψ^	Plaque volume (mean ± SE, mm^3^) at 1-mo scan	Plaque volume (mean ± SE, mm^3^) at 12-mo scan	Volume variation (%) between scans	p-value
**Pre-stent**	29.9 ± 2.2	27.7 ± 2.2	-7	0.307
**Proximal intra-stent tertile**	31.7 ± 2.4	32.0 ± 2.9	1	0.920
**Middle intra-stent tertile**	31.5 ± 2.3	29.7 ± 2.4	-6	0.272
**Distal intra-stent tertile**	29.1 ± 1.8	26.7 ± 2.4	-8	0.155
**Post-stent**	24.0 ± 2.0	23.0 ± 2.0	-4	0.915

^ψ^*A total of 27 patients*, *36 stents and 309 blocks were analyzed*. *Nomenclature*: *there are 3 intra-stent tertiles*. *First tertile lies distally adjacent to the pre-stent block*. *Last (3rd) tertile lies proximally adjacent to the post-stent block*. P-values are obtained by the spline regression multivariate analysis.

#### LAP volume analysis

*LAP volume using the 5mm-long blocks model of segmentation*. Table 8 shows mean LAP volume of pre-, intra- and post-stent blocks at 1- and 12-month follow-ups, according to the 5mm-long model of plaque segmentation. There is no significant change in LAP volume from 1- to 12-month follow-up in all locations.

**Table 8:** Absolute LAP volume at 1- and 12-month follow-up by block location (pre-, intra 1-, 2-, 3-, 4-, 5-, and post-stent blocks).

Block location^ψ^	LAP volume (mean ± SD, mm^3^) (# blocks) at 1-mo scan	LAP volume (mean ± SD, mm^3^) (# blocks) at 12-mo scan	Volume variation (%) between scans	Interfollow-up p-value
**Pre-stent**	7.88 ± 4.66 (28)	6.09 ± 2.61 (18)	-23	0.139
**Intra-stent 1**	8.09 ± 5.74 (33)	8.13 ± 4.29 (25)	0.5	0.866
**Intra-stent 2**	7.77 ± 4.87 (34)	7.47 ± 5.26 (26)	-4	0.861
**Intra-stent 3**	6.38 ± 2.70 (30)	6.85 ± 4.37 (24)	7	0.336
**Intra-stent 4**	7.14 ± 2.52 (14)	7.68 ± 4.84 (12)	8	0.385
**Intra-stent 5**	6.30 ± 3.04 (9)	5.76 ± 2.14 (7)	-9	0.995
**Post-stent**	6.35 ± 3.93 (29)	6.16 ± 4.13 (20)	-3	0.997

^ψ^A total of 27 patients, 36 stents and 309 blocks were analyzed

Note: The intra-stent blocks are enumerated from proximal to distal edge of the stent. Some stents are only 1-,2-,3-,4-blocks long (see stent dimensions in Table 5). P-values are obtained by the ANOVA analysis with Bonferroni correction.

*LAP volume using the tertiles model of segmentation*. Table 9 demonstrates LAP volume in the pre-, intra- and post-stent locations after 1- and 12-month scans, using the tertile model of segmentation for intra-stent plaque. There is no significant change in LAP volume from 1- to 12-month follow-up in all locations.

**Table 9:** Absolute LAP volume at 1- and 12-month follow-up by plaque location (pre-, 1^st^, 2^nd^, 3^rd^ intra-stent tertiles, and post-stent blocks).

Plaque location^ψ^	LAP volume (mean ± SE, mm^3^) at 1-mo scan	LAP volume (mean ± SE, mm^3^) at 12-mo scan	Volume variation (%) between scans	p-value
**Pre-stent**	7.7 ± 0.8	6.6 ± 0.7	-13	0.305
**Proximal intra-stent tertile**	8.0 ± 1.1	7.6 ± 0.9	-6	0.600
**Middle intra-stent tertile**	7.4 ± 0.8	7.2 ± 0.9	-2	0.827
**Distal intra-stent tertile**	6.4 ± 0.6	7.0 ± 1.0	8	0.527
**Post-stent**	6.3 ± 0.7	6.2 ± 0.9	-2	0.749

^ψ^*A total of 27 patients*, *36 stents and 309 blocks were analyzed*. *Nomenclature*: *there are 3 intra-stent tertiles*. *First tertile lies distally adjacent to the pre-stent block*. *Last (3*^*rd*^*) tertile lies proximally adjacent to the post-stent block*. P-values are obtained by the spline regression multivariate analysis.

#### Relative LAP (%) analysis

*Relative LAP volume using the 5mm-long blocks model of segmentation*. Table 10 shows mean ratio of LAP volume over total plaque volume (%) in the pre-, intra- and post-stent blocks after 1- and 12-month follow-up, according to the 5mm-long model of plaque segmentation. There is no significant change in relative LAP volume from 1- to 12 -month follow-up in all locations.

**Table 10:** Relative LAP volume at 1- and 12-month follow-ups by block location (pre-, intra 1-, 2-, 3-, 4-, 5-, and post-stent blocks).

Block location^ψ^	% LAP volume (mean ± SD, %) (# blocks) at 1-mo scan	% LAP volume (mean ± SD, %) (# blocks) at 12-mo scan	Volume variation (%) between scans	Interfollow-up p-value
**Pre-stent**	27.08 ± 12.60 (28)	25.14 ± 9.29 (18)	-7	0.312
**Intra-stent 1**	24.26 ± 12.32 (33)	25.73 ± 10.77 (25)	6	0.640
**Intra-stent 2**	24.59 ± 11.17 (34)	23.67 ± 9.25 (26)	-4	0.606
**Intra-stent 3**	23.14 ± 10.55 (30)	24.94 ± 10.62 (24)	8	0.450
**Intra-stent 4**	24.60 ± 8.18 (14)	27.72 ± 10.69 (12)	13	0.198
**Intra-stent 5**	24.55 ± 10.97 (9)	27.94 ± 10.68 (7)	14	0.389
**Post-stent**	27.88 ± 12.31 (29)	29.01 ± 13.89 (20)	4	0.715

^ψ^A total of 27 patients, 36 stents and 309 blocks were analyzed.

Note: The intra-stent blocks are being enumerated from proximal to distal edge of the stent. Some stents are only 1-,2-,3-,4-blocks long (see stent dimensions in Table 5). P-values are obtained by the ANOVA analysis with Bonferroni correction.

*Relative LAP volume using the tertile model of segmentation*. Table 11 demonstrates mean ratio of LAP volume over total plaque volume (%) in the pre- intra- and post-stent locations after 1- and 12-month scans, using the tertile model of segmentation for intra-stent plaque. There is no significant change in relative LAP volume from 1- to 12-month follow-up, although the slight increase in the distal intra-stent tertile is almost significant (p = 0.052).

**Table 11:** Relative LAP volume at 1- and 12-month follow-up by plaque location (pre-, 1^st^, 2^nd^, 3^rd^ intra-stent tertiles, and post-stent blocks).

Plaque location^ψ^	% LAP volume (mean ± SE, mm^3^) at 1-mo scan	% LAP volume (mean ± SE, mm^3^) at 12-mo scan	Volume variation (%) between scansd	p-value
**Pre-stent**	26.8 ± 2.3	25.2 ± 1.9	-6	0.541
**Proximal intra-stent tertile**	23.3 ± 2.2	23.4 ± 2.1	0.4	0.936
**Middle intra-stent tertile**	24.6 ± 2.0	23.6 ± 1.7	-4	0.594
**Distal intra-stent tertile**	23.4 ± 1.8	26.9 ± 2.2	15	0.052^∞^
**Post-stent**	27.8 ± 2.3	28.8 ± 2.8	4	0.258

^ψ^*A total of 27 patients*, *36 stents and 309 blocks were analyzed*. *Nomenclature*: *there are 3 intra-stent tertiles*. *First tertile lies distally adjacent to the pre-stent block*. *Last (3rd) tertile lies proximally adjacent to the post-stent block*. P-values are obtained by the spline regression multivariate analysis. ^∞^ = result close to 5% significancy.

There was no interaction between time and zone or block in any previously mentioned ANOVA analysis.

## Discussion

The present study demonstrates the feasibility of noninvasive assessment of intrastent plaque volume and composition after BRS implantation using CT imaging. In this study, we prospectively recruited 27 patients with 36 coronary BRS for repeated assessment of intrastent and extrastent plaque volume and low attenuation component using CT angiography and plaque quantitative volumetric assessment, at 1 and 12 months from stent implantation. All stents were assessable with CT. Serial analyses were performed on 309 five-mm long intrastent and extrastent coronary wall blocks. Results will be discussed subsequently.

Multiple studies have contributed to the growing knowledge on native nonobstructive coronary artery atherosclerosis gained mostly from invasive techniques, namely intravascular ultrasound (IVUS) and optical coherence tomography (OCT), but also from noninvasive CT angiography. In a meta-analysis including 4733 subjects followed up for > 1 year, [17] Bamberg et al. showed that the presence of coronary plaque on CT was associated with a hazard ratio of 4.5 (95% confidence interval 2.2–9.3) for incident events (mostly cardiovascular), i.e. an approximately 4-fold increased risk compared to those without plaques on CT. In addition, more specific CT markers of plaque vulnerability, such as low attenuation plaque [7] and positive remodeling [7, 8], have been associated with increased prevalence [8] and incidence [7] of acute coronary syndrome. An ex vivo human study showed a good accuracy of CT angiography for the detection of coronary plaques with a lipid core, when compared to histological analysis and OCT [18]. In an in vivo study, plaques demonstrating low attenuation plaque and positive remodeling by CT angiography were associated with thin-cap fibroatheroma with macrophage infiltration on OCT [19].

In patients with coronary stents, coronary plaque imaging before as well as after stent implantation is also an important issue. In patients with metallic stents, intrastent plaque imaging is performed using intracoronary procedures (IVUS, OCT) [9–12]. In patients with metallic coronary stents, residual or growing intrastent plaque is directly adjacent to the blooming artifact from the struts and consequently, is not assessable in most cases.

In the ABSORB A Cohort trial, 25 patients with BRS were assessed using CT after 18-month follow-up [20], then reassessed at 60-month follow-up [14, 20]. The authors analyzed intrastent lumen patency, and they were able to assess intrastent lumen area and lumen volume. They also assessed intrastent atherosclerotic plaque volume [14]. A case report also showed that newer magnesium-based BRS could accurately be located using CT during follow-up, after scaffold strut resorption [21]. As shown in the present study, as well as in the study by Campos et al. [14], the BRS with metal-free scaffolds enable plaque CT imaging after stent implantation.

Our study used a prospective design and assessed intra- and extra-stent plaque volume in 27 patients after BRS implantation. In contrast to the study of Campos et al. [14], and for the first time, to our knowledge, we were also able to quantitatively assess, in addition to total plaque volume, the intrastent low attenuation plaque component.

### Results explanation

#### Plaque volume and Absolute LAP volume

In our CT study, plaque volumes in pre- intra- and post-stent were found to be similar at 1 and 12-month follow-ups. There is no established consensus in the literature as to how plaque area or volume evolves over time under the bioresorbable stent. Studies of plaque assessment under and juxta bioresorbable stent are mostly done with IVUS with follow-up ranging from 6 months to 5 years and portray contradictory results [14, 22–24]. Further CT studies are required to infirm or confirm our results.

#### Relative LAP volume

Our study has also demonstrated that relative LAP increases (p = 0.052) in the distal intrastent tertile over time. In a study by Gogas et al. [25], patients with bioresorbable stents were investigated at 6 months (45 patients) and at 1-year (56 patients) follow-ups with virtual histology intravascular ultrasound (VH-IVUS) imaging. Gogas’ study demonstrated that at 1-year post-implantation, there was an increase in the relative fibrofatty tissue in 5-mm distal edge of the stent by 43.3% (p < 0.05) [25].

Of note, Pendyala et al. performed on 8 farm pigs with implanted 18 DES stents, superoxide production was found to be 55% more prominent in four distal millimeters rather than 4 proximal millimeters to the stents [26]. As excessive amount of superoxide can oxidize naturally circulating LDL in the blood, Rodríguez et al. states that the plaque formation increases with high oxidized LDL/ LDL ratio [27].

### Clinical relevance

We believe that data obtained from studies assessing noninvasive CT imaging of bioresorbable stents, such as ours, is of particular importance. First, our results confirm that CT appears as an effective noninvasive imaging tool in patients with bioresorbable coronary stents, as shown by few groups up to now [14, 20]. Second, two recent meta-analyses [28, 29] showed ABSORB bioresorbable coronary stents to be associated with increased risk of stent thrombosis and target lesion failure (target vessel myocardial infarction and lesion revascularisation), in comparison to cobalt-chromium everolimus-eluting metallic stents. Despite these results, improvements can be expected from newer scaffold designs with thinner struts (125 μm and less) [30, 31], eluting drugs other than everolimus [32], dedicated implantation protocols [13], optimized lesion and patient selection, as well as modified antithrombotic therapy protocols [33]. In this context, feasibility of intrastent CT imaging makes the latter a valuable noninvasive tool for assessment of new BRS technologies.

### Strengths and limitations

Our proof-of concept study is the first to analyze the relationship and progression of the intra- and juxta-BRS plaque and LAP volumes by CT scan. Nonetheless, our results should be hypothesis-generating as to the plaque progression mechanisms given the small sample size of our study.

Also, high-risk plaque features, other than its low attenuation component, were not assessed, such as positive remodeling. Furthermore, our CT findings (with one exception) were not correlated with invasive imaging, such as IVUS or OCT.

## Conclusion

Our study confirms the feasibility of the repeated non-invasive quantitative assessment of the intrastent and juxta-stent coronary plaque by CT scan, in patients with bioresorbable stents. The developed method could be applied for the evaluation of different scaffold designs or pharmacological profiles of bioresorbable stents.

## Supporting information

S1 Data (XLSX)
